# Echinocandin persistence directly impacts the evolution of resistance and survival of the pathogenic fungus *Candida glabrata*

**DOI:** 10.1128/mbio.00072-24

**Published:** 2024-03-19

**Authors:** Amir Arastehfar, Farnaz Daneshnia, Daniel J. Floyd, Nathan Elias Jeffries, Mostafa Salehi, David S. Perlin, Macit Ilkit, Cornelia Lass-Flöerl, Michael K. Mansour

**Affiliations:** 1 Division of Infectious Diseases, Massachusetts General Hospital, Boston, Massachusetts, USA; 2 Department of Medicine, Harvard Medical School, Boston, Massachusetts, USA; 3 Institute of Biodiversity and Ecosystem Dynamics (IBED), University of Amsterdam, Amsterdam, The Netherlands; 4 Center for Regenerative Medicine, Boston, Massachusetts, USA; 5 Department Industrial Engineering Faculty of K.N., Toosi University of Technology, Tehran, Iran; 6 Center for Discovery and Innovation, Hackensack Meridian Health, Nutley, New Jersey, USA; 7 Hackensack Meridian School of Medicine, Nutley, New Jersey, USA; 8 Georgetown University Lombardi Comprehensive Cancer Center, Washington, District of Columbia, USA; 9 Division of Mycology, Faculty of Medicine, University of Çukurova, Adana, Türkiye; 10 Medical University Innsbruck, Institute of Hygiene and Medical Microbiology, Innsbruck, Austria; Yonsei University, South Korea

**Keywords:** echinocandin, tolerance, persistence, *Candida glabrata*, *in vivo*, *ex vivo*

## Abstract

Recent epidemiological studies documented an alarming increase in the prevalence of echinocandin-resistant (ECR) *Candida glabrata* blood isolates. ECR isolates are known to arise from a minor subpopulation of a clonal population, termed echinocandin persisters. Although it is believed that isolates with a higher echinocandin persistence (ECP) are more likely to develop ECR, the implication of ECP needs to be better understood. Moreover, replacing laborious and time-consuming traditional approaches to determine ECP levels with rapid, convenient, and reliable tools is imperative to advance our understanding of this emerging concept in clinical practice. Herein, using extensive *ex vivo* and *in vivo* systemic infection models, we showed that high ECP isolates are less effectively cleared by micafungin treatment and exclusively give rise to ECR colonies. Additionally, we developed a flow cytometry-based tool that takes advantage of a SYTOX-based assay for the stratification of ECP levels. Once challenged with various collections of echinocandin-susceptible blood isolates, our assay reliably differentiated ECP levels *in vitro* and predicted ECP levels in real time under *ex vivo* and *in vivo* conditions when compared to traditional methods relying on colony-forming unit counting. Given the high and low ECP predictive values of 92.3% and 82.3%, respectively, our assay showed a high agreement with traditional approach. Collectively, our study supports the concept of ECP level determination in clinical settings and provides a robust tool scalable for high-throughput settings. Application of this tool facilitates the interrogation of mutant and drug libraries to further our understanding of persister biology and designing anti-persister therapeutics.

**IMPORTANCE:**

*Candida glabrata* is a prevalent fungal pathogen able to replicate inside macrophages and rapidly develop resistance against frontline antifungal echinocandins. Multiple studies have shown that echinocandin resistance is fueled by the survival of a small subpopulation of susceptible cells surviving lethal concentrations of echinocandins. Importantly, bacterial pathogens that exhibit high antibiotic persistence also impose a high burden and generate more antibiotic-resistant colonies. Nonetheless, the implications of echinocandin persistence (ECP) among the clinical isolates of *C. glabrata* have not been defined. Additionally, ECP level determination relies on a laborious and time-consuming method, which is prone to high variation. By exploiting *in vivo* systemic infection and *ex vivo* models, we showed that *C. glabrata* isolates with a higher ECP are associated with a higher burden and more likely develop echinocandin resistance upon micafungin treatment. Additionally, we developed an assay that reliably determines ECP levels in real time. Therefore, our study identified *C. glabrata* isolates displaying high ECP levels as important entities and provided a reliable and convenient tool for measuring echinocandin persistence, which is extendable to other fungal and bacterial pathogens.

## INTRODUCTION

*Candida glabrata*, also known as *Nakaseomyces glabratus* according to updated nomenclature (1), is a component of the human gastrointestinal (GI) tract microbiome and is one of the major fungal pathogens counted as the second leading cause of candidemia in the US, Canada, Australia, and some European and Asian countries (2–4). Unlike other *Candida* species, *C. glabrata* is genetically closer to the baker’s yeast, *Saccharomyces cerevisiae*, and is able to survive and replicate within macrophages (5). Alarmingly, an increasing number of studies have noted a relatively high percentage of fluconazole- and echinocandin-resistant (ECR) *C. glabrata* isolates. Notably, some clinical centers have noted that >30% of ECR *C. glabrata* blood isolates are, in fact, multidrug resistant (6–8). ECR typically occurs through the acquisition of mutations in short stretches of the catalytic subunit of β1,3-glucan synthase, known as hotspot 1 (HS1) and HS2 of *FKS1* and *FKS2* (3, 9). Given that echinocandins are the only fungicidal antifungal against *Candida* infection with favorable safety profiles, the cytotoxicity issues associated with polyenes, and the high tolerance and resistance of *C. glabrata* to azoles, the increasing ECR rate could potentially pose a serious threat to clinical management (3, 9). All these observations indicate that *C. glabrata* has a versatile genetic wiring to not only effectively establish systemic infection and cope with stress constraints imposed by the host but also swiftly adapt to currently available systemic antifungal drugs. Accordingly, the World Health Organization has officially declared *C. glabrata* among the fungal pathogens with high priority (10).

Although antifungal resistance has been historically noted as the only clinically important phenomenon, recent studies have proposed that antifungal tolerance and persistence are potentially associated with clinical importance. We previously showed that *C. parapsilosis* mutants with higher ECP levels had a significantly higher ECR relative to parental wild-type strain (11). Similarly, *Candida* isolates displaying high levels of azole tolerance are associated with therapeutic failure in various *in vivo* models and infected patients (12, 13). Interestingly, a recent study demonstrated that mutants with a lower level of echinocandin tolerance, which lacked genes involved in the cell wall integrity pathway (derived from ATCC 90030), also had a lower burden and ECR rate in the context of GI tract colonization upon caspofungin treatment. Nonetheless, compared to those of the parental type strains, the minimum inhibitory concentrations (MICs) of caspofungin are several fold lower for the mutants (14) (by definition, tolerance/persistence should not impact the MIC but rather the persistence level) (15). Therefore, the potential implications of ECP levels among echinocandin-susceptible (ECS) clinical isolates of *C. glabrata* using systemic infection mouse models have yet to be determined.

Antifungal resistance is a genetically inherited property that occurs through the acquisition of mutations in drug targets or efflux pumps, which allows the growth of a given isolate in the presence of antifungal concentrations (either fungistatic or fungicidal) higher than the defined clinical breakpoints/MICs. Theoretically, all fungal cells in each resistant isolate should carry the same genetic mutation, and they should lack the subpopulation effect. However, antifungal tolerance is defined as the slow growth of a major subpopulation (5%–90% of the whole population) in the presence of supra-MICs of static antifungal drugs, such as the tolerance of *C. albicans* to fluconazole (15). Nonetheless, antifungal persistence refers to the survival, not slow growth, of a minor subpopulation (<1% of the whole population) in supra-MICs of fungicidal antifungal drugs, such as the micafungin persistent of *C. glabrata* isolates. Such killing dynamics are reflected by a biphasic killing curve, a hallmark of antimicrobial persisters, where rapid cell death of the majority of a susceptible clonal population of cells is followed by a slow killing rate of persister cells (16). Notably, unlike bacteriology, these concepts are novel phenomenon in medical mycology; therefore, the mechanisms underlying both concepts are yet to be fully understood. The initial findings on both concepts suggest stochastic heterogeneity, such as physiological differences, in a presumably clonal susceptible population (15, 16). Recent studies on *C. glabrata* have identified mitochondrial disruption as one of the mechanisms potentially contributing to echinocandin persistence (17–19). However, aneuploidy has been suggested to be a mechanism potentially driving fluconazole tolerance (20).

In fact, determination of antifungal resistance using internationally standardized protocols has been used as the cornerstone of patient care. Similarly, Berman’s group recently described a rapid and convenient approach for determining the azole tolerance in *Candida* species (12). In contrast, ECP level determination still relies on the traditional labor-intensive, time-consuming, and expensive colony-forming unit (CFU) counting approach. This issue could be further exacerbated by the slow growth of growth-arrested persisters upon transfer to drug-free agar plates, which sometimes require 72 hours of incubation. Moreover, the tiny population of persisters, which also has downregulated respiration, challenges the applicability of tools relying on adenosine triphosphate measurements as a readout, which could be successfully used to discern susceptible and resistant isolates (21). Finally, since persisters encompass an extremely low subpopulation whose size varies depending on the drug concentration and exposure time and because they do not replicate, antifungal susceptibility testing protocols are unable to infer the persistence level (14, 17, 18). Therefore, devising a rapid, convenient, and inexpensive method capable of reliably predicting persister levels in real time could enhance advancements in various aspects of persister biology in mycology and microbiology in general, such as high-throughput mutant library screening aimed at identifying novel drug targets and screening drug libraries exerting anti-persister activities.

Herein, we used comprehensive *in vitro*, *ex vivo*, and *in vivo* models to assess the association between the ECP level of collection of clinical *C. glabrata* isolates displaying high and low ECPs (HP and LP, respectively) with burden and the ECR colony rate. Our *ex vivo* model employing primary human monocyte-derived macrophages (hMDMs) treated with micafungin revealed that ECR isolates only occur among HP isolates, which generally also had a higher burden. Similarly, mice infected with HP isolates exclusively had a higher burden in all organs tested and harbored ECR colonies. Moreover, we developed a rapid flow cytometry-based approach that reliably determined the level of ECP *ex vivo* and *in vivo* compared to the traditional CFU-based method. Our study showed that the ECP level is an important fungal entity as HP isolates have a significantly higher burden and ECR rate *in vivo*. Moreover, our flow cytometry-based assay can be used as a high-throughput method and can be applied to various fungal pathogens to determine ECP rapidly and conveniently for mutant and drug library screening purposes.

## RESULTS

### Macrophages infected with high-persister *C. glabrata* isolates have a higher burden and ECR rate upon treatment with micafungin

**Fig 1:**
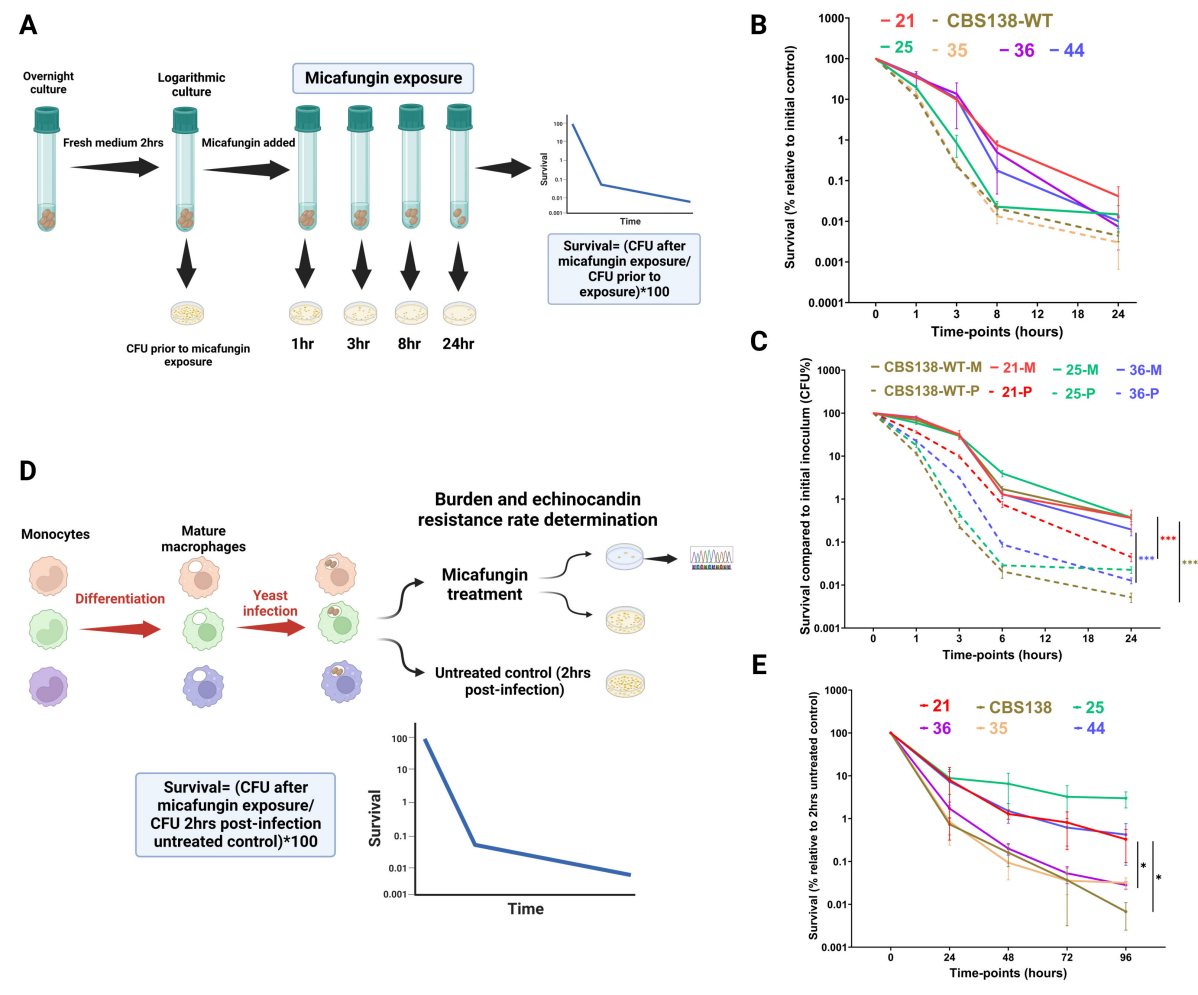
High-persister *C. glabrata* isolates exhibited increased micafungin tolerance both *in vitro* and *ex vivo* (21 and 25 as high persisters, 36 and 44 as unstable high persisters, whereas CBS138 and 35 were noted as low persisters). Overnight-grown *C. glabrata* isolates were incubated in cRPMI medium for 2 h, followed by exposure to micafungin (0.125 µg/mL), and survival was evaluated at different timepoints during the course of 24 h of exposure (**A**). The *in vitro* killing dynamics of various echinocandin-susceptible *C. glabrata* isolates treated with micafungin (0.125 µg/mL) revealed variation in echinocandin persistence (ECP) (**B**). Individual comparisons between CBS138 (a low persister) and other isolates and pertinent statistical analysis are presented in Fig. S1. The micafungin tolerance of *C. glabrata* isolates with varying ECP levels was equal after 2 h of internalization by macrophages (denoted as M), whereas the same isolates actively growing in cRPMI show significantly lower ECP levels (denoted as P) (**C**). Mature human primary macrophages were obtained from the primary human monocytes of three healthy donors. After maturation by incubation in macrophage colony stimulating factor, primary human macrophages were infected with the various *C. glabrata* isolates. The macrophages were extensively washed 2 h post infection and treated with fresh cRPMI with or without micafungin (0.125 µg/mL), after which the killing dynamics were monitored for 5 days. The burden was calculated by normalizing the CFU of treated wells against the intracellular untreated control at 1 h post infection. The emergence of echinocandin-resistant colonies was determined by plating treated samples on agar plates containing micafungin (0.125 µg/mL), followed by *FKS* mutation characterization (**D**). Macrophages infected with high persister isolates generally had a higher burden than did their low-persister counterparts (**E**). Notably echinocandin resistance was only noted for two of the high-persister isolates (21 and 25), not low persisters. At each timepoint, at least three biological replicates were included in panels B and C, and panel D shows the macrophage data of at least three healthy donors. Two-tailed *t*-test was used for statistical analysis, and values ≤0.05 were considered to indicate statistical significance. Symbols *, **, and *** indicate *P*-values ≤0.05, <0.01, and <0.001, respectively.

To probe the impact of the echinocandin persistence level on survival and the ECR rate, we used six *C. glabrata* isolates, including the original genome-sequenced strain CBS138 and five clinical isolates, which had the same micafungin MIC values (Table S1). We inoculated 10^7^ CFU of those isolates in complete RPMI [(cRPMI, 1% L-glutamine, 1% pen-strep, and 10% heat-inactivated phosphate-buffered saline (FBS)] medium supplemented with micafungin (0.125 µg/mL) and monitored survival at 1, 3, 8, and 24 h post exposure (pe) by plating and CFU counting (Fig. 1A). The HP designation was based on statistical difference of CFU values between a given isolate and the type strain CBS138 at 24 h. More specifically, isolates exhibiting a significantly higher CFU at 24 h were considered as HP (21 consistently HP at all timepoints and 25 only at 24 h), whereas those showing initial high CFU, followed by a decline at 24 h, were considered unstable HP isolates (36 and 44). We also noted that isolate 25 showed an initial low persistence phenotype, followed by an HP phenotype at 24 h, whereas isolate 35 appeared to have CFU values similar to those of CBS138 and thus was designated as LP (Fig. 1A and B; Fig. S1). Therefore, this collection featured a viable collection of *C. glabrata* isolates encompassing a wide range of ECP levels while displaying the same micafungin MIC values.

We have previously shown that brief internalization of CBS138 by THP1 macrophages for 3 h dramatically increases the ECP level by enriching persisters (18). Therefore, we hypothesized that internalization by fully mature hMDMs would enrich the persisters in all *C. glabrata* isolates, and thereby, they would exhibit similar survival regardless of the ECP level upon exposure to cidal concentrations of micafungin (hereafter refer to as hMDMs). *C. glabrata* isolates were cocultured with hMDM for 2 h, and following extensive washing to eliminate the non-engulfed *C. glabrata* cells, the macrophages were lysed, the *C. glabrata* cells were collected and exposed to cRPMI containing micafungin (0.125 µg/mL) and survival was monitored at 1, 3, 8, and 24 h post exposure Survival was inferred by normalizing CFU at each time point to the initial CFU prior to micafungin treatment. Interestingly, in line with our expectations, all isolates showed similar ECP levels upon short-term macrophage internalization, and consistent with our previous observations, the ECP levels of the macrophage-internalized isolates were significantly higher than the ECP levels of their counterparts observed *in vitro* (Fig. 1C).

Next, we wondered whether macrophages infected with HP or LP would have different burdens and ECR rates upon long-term co-culture (up to 96 h) and micafungin treatment (0.125 µg/mL). Therefore, upon co-culture for 2 h, the macrophages were extensively washed with PBS and treated with cRPMI supplemented with micafungin (0.125 µg/mL), and macrophages were lysed with cold water at designated timepoints (24, 48, 72, and 96 h), with a portion of the lysate transferred to drug-free agar plates and the other portion was transferred to micafungin-containing agar plates (0.125 µg/mL) to assess burden and ECR rate, respectively. Survival was reported by normalization of the burden of treated macrophages at each timepoint against the burden of untreated macrophages 2 h post infection (pi) (Fig. 1D). The colonies growing on micafungin-containing plates were further subjected to sequencing of HS1 and HS2 of *FKS1* and *FKS2*. Notably, our analysis did not include the burden and ECR rate in cRPMI alone since we previously observed phenotypic resistance and extremely low levels of *bona fide* ECR colonies harboring mutations in the HS regions of the *FKS1* and *FKS2* genes (18). For scientific rigor, we used only fully mature macrophages derived from the monocytes of three healthy donors. Interestingly, consistent with the *in vitro* experiments, the LP isolates maintained low ECP levels at all time points, and except for one of the HP isolates (isolate 36), the remaining HP isolates had a significantly higher burden at all time points tested (Fig. 1E). Intriguingly, compared with those of THP1-derived macrophages, primary macrophages harbored significantly lower ECR colonies, and only HP isolates produced ECR colonies (Fig. 1E; Table 1) (18). Collectively, these observations suggested that although short-term macrophage internalization equalizes the ECP level, HP isolates generally have a significantly higher burden and ECR rate upon long-term macrophage internalization. As such, the ECP level may predict the burden and ECR emergence after micafungin treatment.

**TABLE 1:** Echinocandin-resistant colonies emerged from primary human macrophages^*a*^

Isolate number	Donors	Mutation type	Mutational frequency	Timepoint
21	Donor 1	S663F	0.040816327	96 h
	Donor 2	S663F	0.000408163	72 h
	Donor 3	None	None	None
25	Donor 1	D665Y	0.00027972	72 h
	Donor 2	None	None	None
	Donor 3	None	None	None

^*a*^ Mutational frequency was measured by normalizing the number of ECR colonies against the total colony-forming units of pertinent treated macrophages.

### Systemic infection with the HP isolate, but not the LP isolate, induces a significantly higher burden and ECR rate upon treatment with micafungin *in vivo*

Notably, multiple recent studies have suggested GI tract as the significant source of ECR (22, 23). Nonetheless, upon systemic infection, *C. glabrata* travels to multiple organs. Additionally, given that echinocandins differentially penetrate various organs (24), it is plausible to assume that ECR colonies can also emerge from a given niche and potentially spread to other organs.

**Fig 2:**
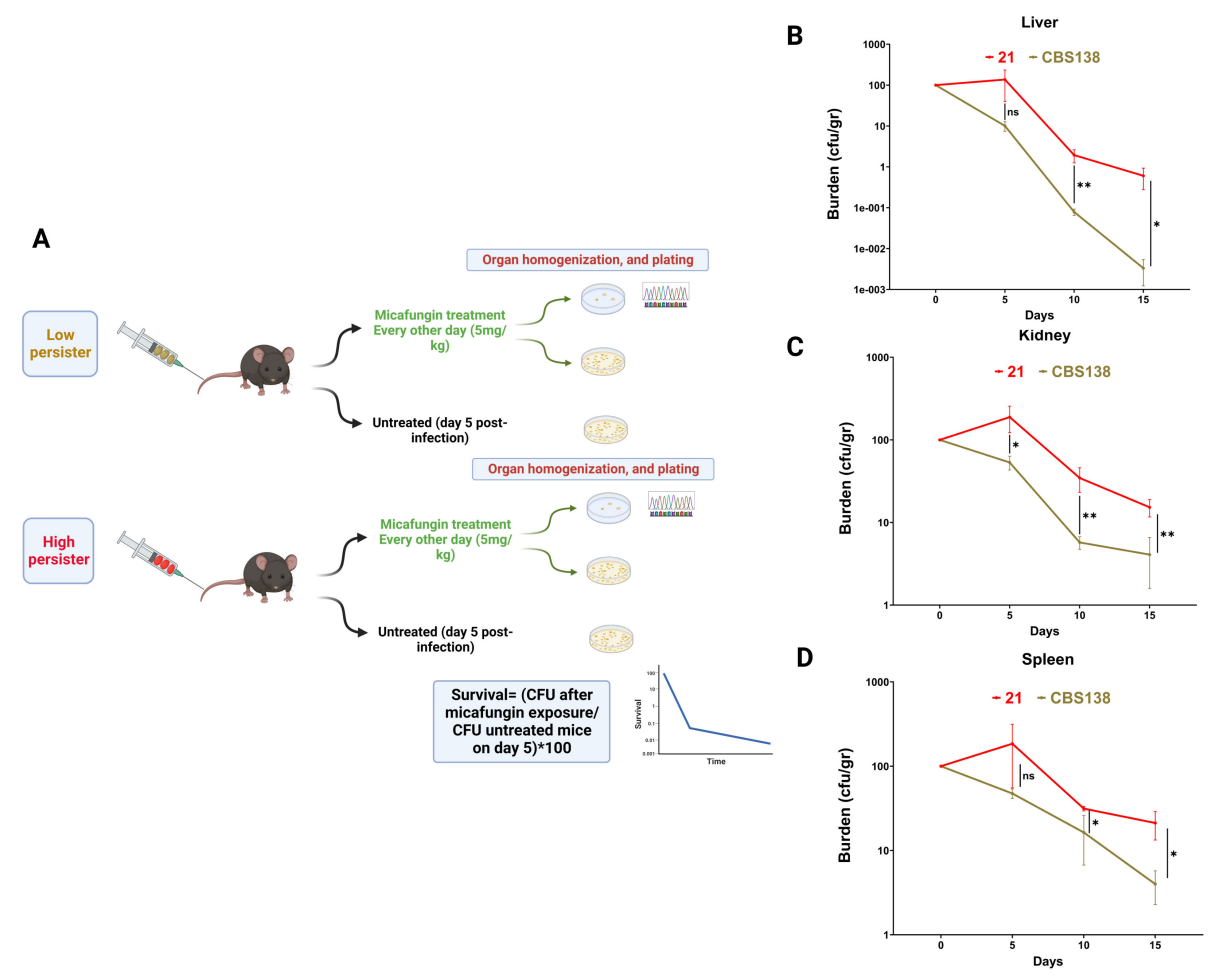
Mice infected with a high-persister isolate (21) had a significantly higher burden and exclusively harbored echinocandin-resistant colonies after treatment with humanized dose of micafungin (5 mg/kg). Mice were grouped into two major groups (26 mice in total, 13 mice per group); infected with high (isolate 21) or low (CBS138) persister *C. glabrata* isolates treated (10 mice for each isolate, 3 mice for days 5, 3 mice for 10 days, or 4 mice for 15 days) or untreated (only three mice for 5 days per isolate) subgroups. Micafungin treatment (5 mg/kg) was initiated 1 day after infection and continued every other day throughout the end of the experiment. Mice were sacrificed at designated timepoints, and the kidneys, liver, and spleen were homogenized and plated on agar plates with or without micafungin to determine the echinocandin resistance rate and burden, respectively. The burden was defined by normalizing the CFU of treated mice against that of untreated mice (**A**). Mice infected with the high-persister (isolate 21) isolate had significantly higher burdens in the liver (**B**), kidney (**C**), and spleen (**D**). Two-tailed *t*-tests were used for statistical analysis; values ≤0.05 were considered to indicate statistical significance. Symbols *, **, and *** indicate *P*-values ≤0.05, <0.01, and <0.001, respectively.

Inspired by our previous observations, we hypothesized that HP isolates could also have a significantly higher burden and ECR rate than LP isolates in the context of *in vivo* systemic infection. Therefore, we selected two isolates, one exhibiting stable HP (isolate 21) and one exhibiting stable LP (CBS138) and induced systemic infection in immunocompetent C57BL/6 mice via tail vein injection (see Materials and Methods). Mice were grouped into two major arms, infected with HP or LP, and each major arm had two subgroups, untreated and treated with a humanized dose of micafungin (5 mg/kg) (24). At the designated timepoints, the kidney, liver, and spleen were harvested and homogenized; a portion was plated on a drug-free agar plate; and another portion was transferred onto micafungin-containing agar plates (0.125 µg/mL) to determine the burden and ECR rate, respectively. Burden was determined by normalization of the CFU of treated mice at each timepoint (days 5, 10, and 15 pi) against that of untreated mice on day 5 (Fig. 2A). In general, the burden was the highest in the spleen, followed by the kidney, and the liver had the least burden. In fact, this observation is in line with a previous study documenting liver and kidney containing the highest and the lowest micafungin concentrations from autopsy samples, respectively (25). Similarly, we previously showed that caspofungin, not micafungin, had a lower concentration in the spleen but still higher than the MIC, compared to kidney (18).

Interestingly, mice infected with the HP isolate had a significantly higher burden at almost all timepoints and organs (Fig. 2B through D). Next, we asked whether mice infected with the HP isolate also had more ECR colonies. Intriguingly, ECR colonies were exclusively observed for mice infected with the HP isolate but not for those infected with the LP isolate. Reflecting the observation of the lower burden of liver and kidney compared to spleen, the vast majority of the ECR colonies were detected in these two organs. This may explain why a higher selective pressure, albeit tolerable by *C. glabrata*, can result in a higher frequency of ECR mutants, which is similar to what has been observed in GI tract mice models, where ECR colonies were significantly higher for mice treated with high dosages of caspofungin (23). All ECR colonies harbored mutations in HS1 of Fks2 (Table 2).

**TABLE 2:** Echinocandin-resistant colonies obtained from isolate 21 in various organs post infection from mice systemically infected with *C. glabrata^a^*

Days post infection	Fks amino acid substitution			Mutational frequency		
	Kidney	Liver	Spleen	Kidney	Liver	Spleen
Day 5	TBD	F659del	None	6.94E-06	3.28E-05	0
	TBD	None	None	6.67E-06	0	0
	R665S and F659del	S663F	None	8.89E-06	1.75E-05	0
Day 10	L714F	None	F659del and P667H	0.000262	0	0.000128
	None	F659del	None	0	0.001564	0
	None	None	None	0	0	0
Day 15	None	None	None	0	0	0
	None	None	None	0	0	0
	None	None	TBD	0	0	6.18E-05
	None	None	None	0	0	0

^*a*^ Mutational frequency was measured by normalizing the number of ECR colonies against the total colony-forming units of pertinent treated mice. Each row in each day represents one mouse (for instance, three mice harbored ECR colonies in different organs).

These observations collectively suggested that infection with HP isolates results in a significantly higher burden and number of ECR colonies in the context of systemic infection mouse model. As such, ECP level *in vitro* can potentially predict the likelihood of a higher burden and a higher number of ECR mutants *in vivo*.

### A rapid SYTOX-based assay using flow cytometry can precisely predict ECP levels in real time

**Fig 3:**
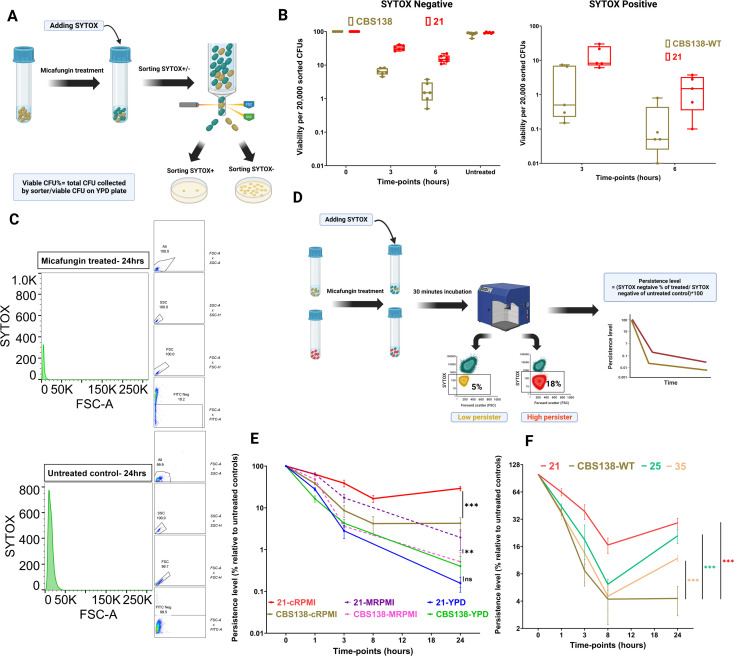
The echinocandin persistence (ECP) level can be precisely determined by a SYTOX-based flow cytometry assay. High and low echinocandin persister isolates were exposed to a cidal concentration of micafungin (0.125 µg/mL), stained with SYTOX, and subjected to fluorescent activated cell sorting. A total of 20,000 SYTOX− and SYTOX^+^ CFUs were collected and plated to determine the viability at each timepoint (**A**). The viability of SYTOX^+^ plants was significantly lower than that of SYTOX^−^ counterparts, and the viability dramatically decreased at a later timepoint. Moreover, the SYTOX^−^ events collected from high persisters had significantly higher viability compared to the lower persister ones (**B**). The gating strategy used to differentiate the SYTOX^−^ fraction of *C. glabrata* isolates (**C**). The schematic workflow for distinguishing the ECP level using SYTOX-based flow cytometry (**D**). The ECP level was more discriminatory when the incubation with the drug was carried out in MOPS-treated RPMI and cRPMI (**E**). Subjecting the initial collection of *C. glabrata* isolates to micafungin in cRPMI supplemented with micafungin (0.125 µg/mL) readily differentiated the high and low micafungin persisters (**F**). Two-tailed *t*-test was used for statistical analysis, and values ≤0.05 were considered to indicate statistical significance. Symbols *, **, and *** indicate *P*-values ≤0.05, <0.01, and <0.001, respectively.

Now that we have shown that ECP level matters as validated by *ex vivo* and *in vivo* models and given the issues associated with the traditional CFU counting approach, we sought to develop a precise, convenient, and inexpensive method to rapidly determine ECP levels in real time. Cell-impermeable dyes, such as SYTOX and propidium iodide, are extensively used for various biological functions to determine viability. Inviable cells lose their membrane integrity, and once exposed to the cell impermeable dyes, the cytoplasm/nucleus will be stained upon translocation of the dye into the cell interior. Since LP isolates are shown to have lower viable CFUs than HP isolates, we hypothesized that upon micafungin exposure, the intensity of a given cell-impermeable dye would be proportional to the number of dead cells. As such, LP isolates will have a higher staining intensity than HP isolates. Therefore, we used SYTOX for staining and determined the staining intensity using flow cytometry to rapidly determine the viability. To prove the applicability of this approach, we incubated HP (isolate 21) and LP (CBS138) in cRPMI supplemented with micafungin (0.125 µg/mL) and cRPMI alone for 3 and 6 h, respectively, followed by SYTOX staining, fluorescence-activated cell sorting of SYTOX^Pos^ and SYTOX^Neg^ events (20,000 events), and plating (Fig. 3A). Interestingly, the viability of the treated SYTOX^Neg^ was significantly higher than that of their SYTOX^Pos^ counterparts. Nonetheless, the viability of the SYTOX^Neg^ events was much less than 100%, and it decreased over time, which is consistent with the “viable but non-culturable notion (VBNC)” phenomenon (26, 27), and the extent of culturability was close to that of the antibiotic persister and echinocandin persister *C. glabrata* cells (18, 28). Apart from dramatic metabolic and physiological changes, the decreased viability may be partly due to the sheer stress applied to SYTOX^Neg^ yeast cells during sorting. Interestingly, the SYTOX^Neg^ HP events had a significantly higher viability than did their LP counterpart (Fig. 3B). Similarly, after flow cytometry, the SYTOX^Neg^ fraction was significantly higher for HP isolate than the LP counterpart at different timepoints (Fig. 3B). This simple experiment suggested that SYTOX^Neg^-based flow cytometry assay could potentially discriminate the ECP level.

Since the laboratory media used in mycological applications have different compositions and sugar levels, which could impact metabolic status and persister level, we sought to identify the best medium with the ability to properly distinguish the persistence level. To this end, we inoculated 10^7^ CFUs of LP and HP strains in yeast-peptone-dextrose (YPD) broth, MOPS-treated RPMI media traditionally used for antifungal susceptibility testing, and cRPMI supplemented with micafungin (0.125 µg/mL), followed by SYTOX staining, flow cytometry, and determination of the SYTOX^Neg^ fraction (Fig. 3C and D). YPD media did not differ between the LP and HP isolates, whereas MOPS-treated RPMI media and, particularly, the cRPMI could precisely distinguish the HP and LP isolates. We continued our experiments with cRPMI since the difference between the LP and HP was significantly higher than the other media (Fig. 3E). The higher predictability power of cRPMI could be due to the inclusion of serum, an important blood component since it binds to micafungin and significantly impacts its activity. Therefore, several studies have suggested/advocated the inclusion of serum in antifungal susceptibility testing to better predict *in vivo* outcomes (29, 30). To ascertain if serum can impact the resolution of a given media in separation of LP and HP isolates, we inoculated the same isolates in MOPS-treated RPMI and YPD supplemented with 10% heat-inactivated PBS and micafungin (0.125 µg/mL) and monitored the SYTOX^Neg^ fraction over the same time periods. Interestingly, the inclusion of PBS not only separated the LP and HP isolates but also increased the SYTOX^Neg^ fraction (Fig. S2A). To assess the reproducibility of our assay, we included both CBS138 and isolate 21; exposed them to micafungin, defined as the SYTOX^Neg^ fraction at 24 h; and determined the standard deviation between replicates on a single day and the average of replicates on different days. We found that the SYTOX^Neg^ fraction of the LP and HP isolates deviated between 9.2 ± 0.3–0.4 and 33 ± 0.7–3.03, respectively, in the same day. However, the SYTOX^Neg^ fraction values measured on different days deviated between 33 ± 0.34 and 9.2 ± 1.4 for the HP and LP isolates, respectively.

Next, we assessed the ability of our assay to predict the ECP levels of aforementioned collection of *C. glabrata* isolates, which were proven to display varying ECP levels through CFU counting. Interestingly, compared with CFU counts, our assay could distinguish the LP and HP isolates (Fig. 3F), albeit with some discrepancies (Fig. S2B). The *in vivo* relevance of this discrepancy will be further discussed in the context of the Austrian collection of *C. glabrata* isolates in the following section. Using this collection of *C. glabrata* isolates, the tentative threshold of a ≥28% SYTOX^Neg^ fraction could distinguish the isolates as LP or HP (Fig. S2B). We also subjected a collection of HP mutants of *C. parapsilosis* derived from ATCC 22019, which were previously validated by CFU counting (11). Consistently, the mutants showed a higher SYTOX^Neg^ fraction compared to the wild-type parental strain (ATCC 22019) (Fig. S2C; Table S2). Overall, our SYTOX-based assay could readily detect ECP levels in various *Candida* species displaying varying ECP levels.

### SYTOX-based flow cytometry assay predicts the *ex vivo* and *in vivo* ECP levels

**Fig 4:**
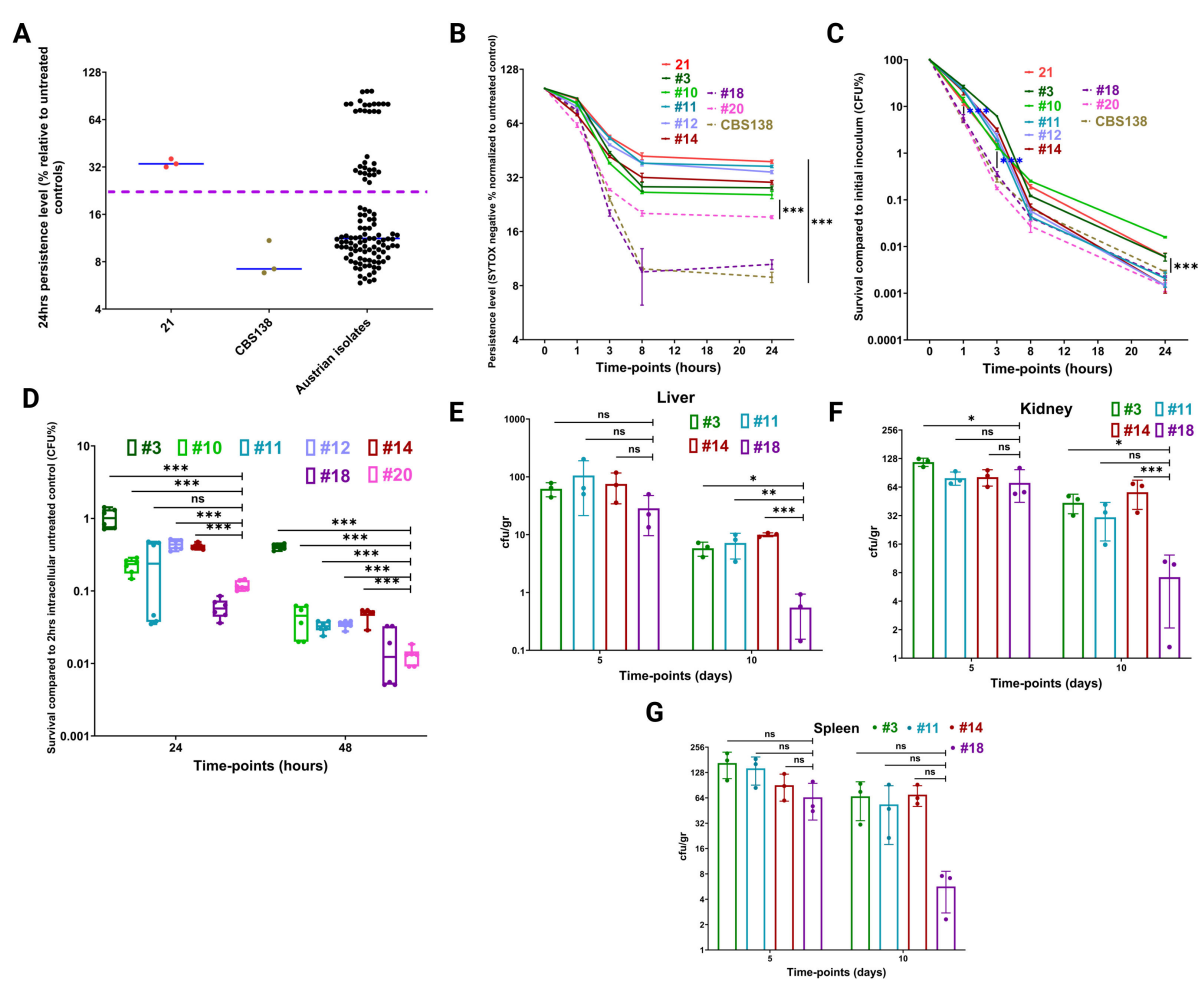
The SYTOX-based assay better predicts micafungin tolerance *ex vivo* and *in vivo*. A collection of echinocandin-susceptible (*n* = 31) and echinocandin-resistant isolates harboring various FKS mutations were subjected to micafungin (1 µg/mL) for 24 h, and their ECP levels were determined by our assay (21 and CBS138 were used as high- and low-persister control isolates, respectively). After setting a threshold, high and low persisters were readily differentiated. Five isolates were high persisters (**A**). The time-kill curve of Austrian high persisters resolutely distinguished high (3, 10, 11, 12, and 14) and low persisters (18 and 20) (**B**), whereas extensive CFU counting showed that some of the high persisters lost their tolerance at later timepoints and, therefore, grouped with low persisters (**C**). All the experiments included at least three biological replicates. All the high persisters (3, 10, 11, 12, and 14) and two randomly selected low persisters (18 and 20) were subjected to *ex vivo* burden measurements following micafungin treatment at 24 and 48 h. Consistent with the time-kill curve of SYTOX-based assay, all the high persisters had a significantly higher intracellular burden (**D**) (Mann-Whitney *U* test). Mice were grouped into two major groups (36 mice in total, 9 mice per group) and infected with high- or low-persister *C. glabrata* isolates, and each group had treated (three mice only for 5 days and three mice for 10 days) or untreated (three mice for only 5 days per isolate) subgroups. Like in the *ex vivo* assay, all the high persisters defined by our SYTOX-based assay, including the unstable persisters defined by CFU counting, were *bona fide* high persisters in the context of the systemic mouse infection model. High Austrian persisters generally had a higher burden in all organs tested, especially at later timepoints (**E and F**) (for all *in vivo* analyses, analysis of variance was performed, except for day 5 liver, which was carried out with the Kruskal-Wallis test). Values ≤0.05 were considered to indicate deemed significant. Symbols *, **, and *** indicate *P*-values ≤0.05, <0.01, and <0.001, respectively.

To further validate the applicability of our assay for discerning the HP and LP isolates, we challenged our assay with a collection of ECS *C. glabrata* blood isolates collected from Innsbruck, Austria (Table S3). Since these ECS isolates had various MICs but below the ECR threshold, we used a micafungin concentration eightfold higher than 0.125 µg/mL (1 µg/mL) to reduce the impact of differences in MIC. All the isolates, including our validated LP and HP strains and six ECR strains harboring various mutations in the HS regions of *FKS1* and *FKS2*, were incubated in cRPMI containing 1 µg/mL, and viability was assessed 24 h after exposure to our SYTOX-based method. Furthermore, we used tentative SYTOX^Neg^ thresholds of ≥28% and <28% to designate the HP and LP isolates, respectively. In total, 16.12% (5/31) of the isolates had a SYTOX^Neg^ fraction equal to or higher than the HP control (threshold of ≥28% SYTOX^Neg^ fraction), and the rest were categorized as LP (Fig. 4A; Table S3). Next, we monitored the SYTOX^Neg^ fraction of all the Austrian HPs (*n* = 5) and randomly selected LPs (*n* = 2) along with our HP and LP control isolates at different timepoints. Interestingly, the HP isolates were further stratified, and all had a significantly higher SYTOX^Neg^ fraction than the LP isolates (Fig. 4B). This stratification was reminiscent of ECR *C. glabrata* isolates harboring various *FKS* mutations and displaying various MIC values, but all categorized as ECR. To validate whether our assay accurately predicted the ECP level, we subjected all these isolates to extensive CFU counting at designated timepoints. Interestingly, similar to our initial observation, the HP isolates formed two different categories; those maintaining the HP phenotype at all timepoints (3 and 10 similar to our HP) and those with an early HP phenotype but declining to the LP level at later timepoints (11, 12, and 14; unstable high persister hereafter) (Fig. 4C). This discrepancy might be due to the VBNC notion and the fact that the lethal concentration of micafungin has impacted major metabolic pathways, rendering a considerable population of the SYTOX^Neg^ fraction unculturable. Nonetheless, this vast VBNC fraction might still have clinical implications unnoticeable by CFU counting. Overall, we developed a convenient, accurate method that could rapidly determine the extent of ECP in a considerable collection of *C. glabrata* isolates in real time.

The discrepancy between our assay and extensive CFU counting prompted us to conduct *ex vivo* and *in vivo* experiments to truly assess the relevance of isolates displaying unstable HPs. To this end, we subjected all Austrian HP (*n* = 2), unstable HP (*n* = 3), and LP (*n* = 2) isolates to our *ex vivo* micafungin treatment model and monitored the burden at 24 and 48 h pi as detailed above. Interestingly, unlike the CFU counting results, the unstable HP isolates had a significantly higher burden in hMDM at both timepoints compared to the LP isolates (Fig. 4D). These results convinced us to run an *in vivo* systemic infection mouse model on one HP (3), two unstable HPs (11 and 14), and one LP (18) to further assess the implications of these stable and unstable phenotypes by measuring burden and ECR rate at 5 and 10 days pi. We used the same setup as mentioned above, i.e., four major groups with two subgroups, treated and untreated. The burden was calculated by normalizing CFU at each timepoint against the CFU of the pertinent untreated group on day 5 pi. The ECR colony rate was monitored by plating on YPD agar plates containing micafungin (0.125 µg/mL). Interestingly, the HP and unstable HP isolates had higher burden across organs and timepoints (Fig. 4E through G). Moreover, ECR colonies were found only for mice infected with the HP and unstable HP isolates and not for those infected with the LP counterpart (Table 3). Like our previous systemic mice infection (animals treated similarly), the majority of the ECR isolates were from the liver, followed by the kidney and spleen. Taken together, our observations indicated that the unstable HP designation found by CFU counting at 24 h does not match the *ex vivo* or *in vivo* results. In contrast, our SYTOX-based assay precisely distinguished the HP and LP isolates.

**TABLE 3:** Echinocandin-resistant colonies obtained from high echinocandin persister isolates from the Austrian collection in various organs post infection from mice systemically infected with *C. glabrata^a^*

Isolates	Days post infection	Fks amino acid substitution			Mutational frequency		
		Kidney	Liver	Spleen	Kidney	Liver	Spleen
3	Day 5				0	0	0
					0	0	0
					0	0	0
	Day 10				0	0	0
		TBD	F659del		0.000679	0.000288	0
					0	0	0
11	Day 5	TBD			0.00004	0	0
					0	0	0
					0	0	0
	Day 10	F659del and TBD			0.001583	0	0
					0	0	0
					0	0	0
14	Day 5	TBD			2.35E-05	0	0
		TBD			0.000119	0	0
		TBD			2.75E-05	0	0
	Day 10			F659del	0	0	0.000234
				F659del	0	0	3.04E-05
					0	0	0

^*a*^ Mutational frequency was measured by normalizing the number of ECR colonies against the total colony-forming units of pertinent treated mice. Each row in each day represents one mouse.

### Three-hour CFU counting and SYTOX-based flow cytometry reliably predict *in vivo* burdens following micafungin treatment

**Fig 5:**
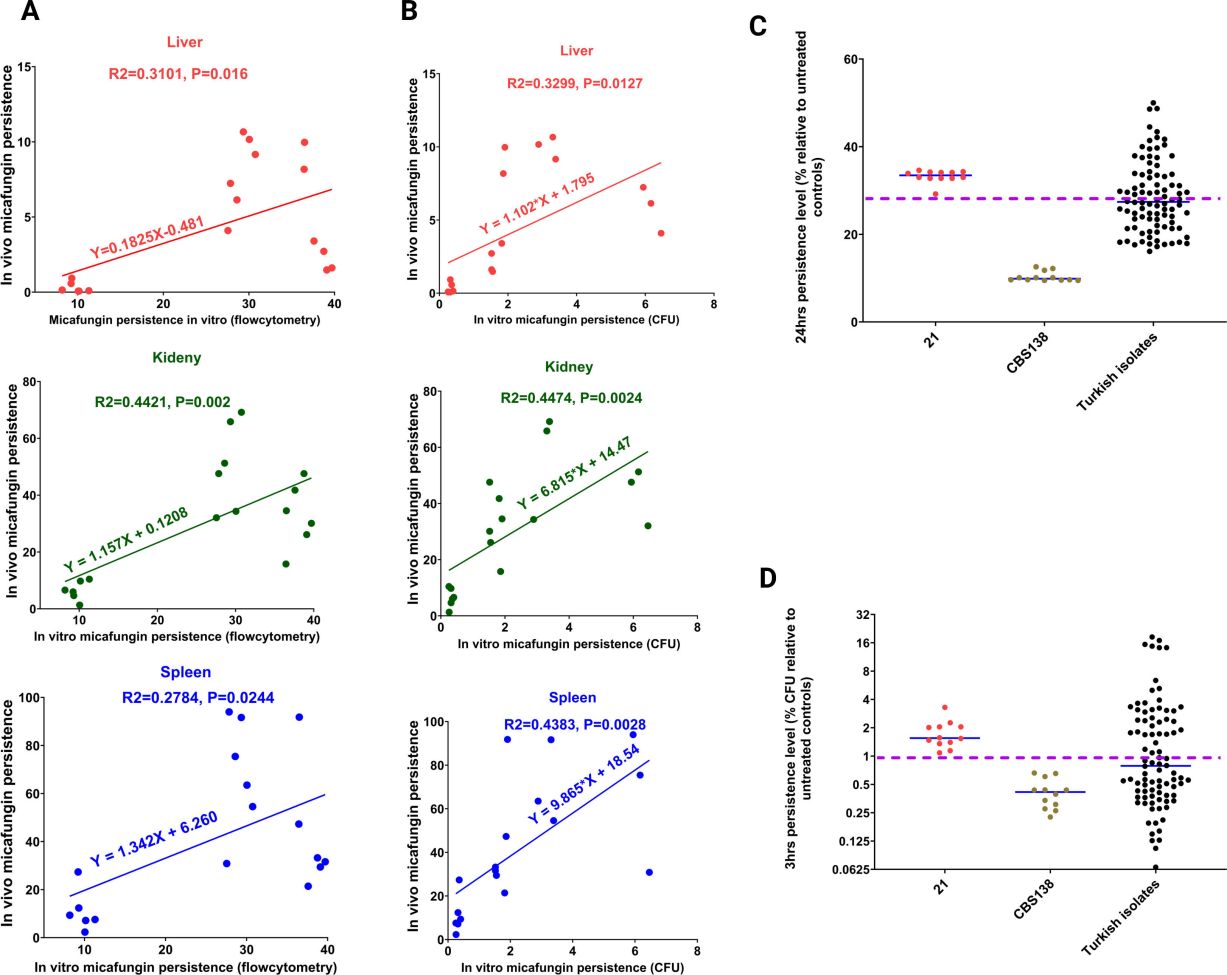
The SYTOX-based assay and 3-h CFU count could reliably predict the *in vivo* burden in different organs. *In vitro* (24-h SYTOX^Neg^ fraction and 3-h CFU counting) and *in vivo* burdens of the HP and LP isolates were used to determine the correlation and regression. Both SYTOX-based assay (**A**) and 3-h CFU counting (**B**) were correlated with *in vivo* burden in all organs tested. Pearson coefficient correlation and simple regression analysis were used. Two-tailed *t*-tests were used for statistical analysis, and values ≤0.05 were considered to indicate statistical significance. The echinocandin persistence of 30 randomly selected *C. glabrata* isolates fully susceptible to all the antifungals was determined using SYTOX-based and 3-h CFU counting assays. Isolates displaying survival ≥1% at 3 h following micafungin exposure (1 µg/mL) (**C**) and SYTOX^Neg^ fractions ≥28% (**D**) were considered HPs. Three independent biological replicates were used for each isolate.

Our previous *in vitro* experiments revealed that CFU counting at 24 h misidentifies unstable HP isolates, i.e., isolates displaying HP and LP phenotypes at 3 and 24 h following micafungin exposure, respectively. Therefore, we examined whether CFU counts at 3 h could reliably predict the *in vivo* burden. Accordingly, we determined the correlation of 3-h CFU counting and our SYTOX-based assay (24 h) with *in vivo* burden on day 10 for mice infected with *C. glabrata* isolates (six isolates: 3, 11, 14, and 18 from the Austrian collection; CBS138; and 21, respectively). Burden on day 10 was chosen since it better dissociated HP and LP isolates than day 5 and that pertinent data were available for both *in vivo* experiments. Once using the 3-h CFU counting, we noted that *C. glabrata* isolates with the survival of ≥1% following micafungin exposure (normalized to CFU prior to micafungin treatment) could reliably distinguish HPs and LP isolates. As such, thresholds of ≥1% and ≥28% were used to distinguish HP from LP isolates using 3-h CFU counting and SYTOX-based method, respectively. Interestingly, both SYTOX-based and 3-h CFU counting were correlated with the *in vivo* burden in all organs tested (Fig. 5A and B). Therefore, 3-h CFU counting could reliably predict *in vivo* burdens and could be used as a benchmark for dissociating LP and HP isolates.

These findings prompted us to further test the SYTOX-based assay in the light of 3-h CFU counting as the gold standard and to systematically determine the HP and LP predictive values. Given that the Austrian *C. glabrata* collection contained a limited number of HP isolates, we examined the persistence of a collection of Turkish *C. glabrata* blood isolates (31). We randomly selected 30 isolates that were fully susceptible to all the tested antifungals and determined the micafungin persistence using 3-h CFU and 24-h SYTOX-based flow cytometry assays. CFU analysis at 3 h identified 17 and 13 isolates as LP and HP, respectively, whereas the SYTOX-based assay identified 15 LP and 15 HP isolates (Fig. 5C and D). Specifically, three and one of the LP and HP isolates, respectively, defined by 3-h CFU were identified as HP and LP, respectively, using a SYTOX-based assay. Therefore, predictive values of the SYTOX-based assay for HP and LP when considering 3-h CFU were 92.3% and 82.3%, respectively. This observation suggested a high degree of agreement between the two methods. Nonetheless, the nature of the controversy for some of these isolates requires future *in vivo* studies to further delineate the distinguishing power of both methods. Taken together, these observations suggest that 3-h CFU counting, but not 24 h, can reliably determine the *in vivo* burden, as can the SYTOX-based assay.

## DISCUSSION

Although the clinical relevance of HP and LP in humans has yet to be validated, we showcased the importance of the ECP level in *C. glabrata* and discovered that isolates with a higher ECP level have a higher burden and ECR rates compared to counterparts with a lower ECP level in the context of a mouse model of systemic infection as well as within primary human macrophages. Accordingly, we developed a reliable method that could accurately predict ECP levels in real time with higher precision than the traditionally used CFU counting. The promising features of our assay make it an ideal fit for high-throughput screening of anti-persister drugs and library of mutants, which also could be extended to other clinically important fungal and even bacterial species. Nonetheless, our assay needs to be examined and validated in other centers and using other collections.

Using an extensive number of clinical *C. glabrata* isolates, we showed that micafungin-treated isolates displaying high ECP levels induced a significantly higher burden in primary human macrophages and mouse models of systemic infection model. Interestingly, although short-term macrophage internalization equally elevated the persistence of all isolates regardless of their basal ECP level, the isolates featuring a higher ECP level had significantly higher survival during long-term internalization, and the survival gap between low and high persisters was extended at later timepoints. Consistently, we detected the ECR colonies only for isolates exhibiting a higher ECP level. These observations fit into the paradigm of bacterial persisters, where the mutational rate and drug resistance are functions of the persister number, i.e., the higher the number of viable persisters, the higher the drug resistance rate (32). Nonetheless, drug resistance can also be dictated by a higher mutational frequency, which was not the scope of the current study. Future studies are required to probe the link between the mutational rate and ECP level and discover the players involved.

Interestingly, we found that the ECR rate was organ dependent, where the most frequently identified mutants were observed for the liver, kidney, and spleen. The difference in the organ-dependent ECR rate could be potentially associated with higher selective pressure, i.e., intra-organ micafungin concentration, as *C. glabrata* burden was significantly lower in the liver and kidney. This observation is consistent with previous studies, where high-dosage caspofungin treatment in GI tract colonized mice not only significantly lowered the abundance of *C. glabrata* in fecal pellets but also dramatically increased the ECR rate compared to counterparts treated with four times less caspofungin (23). Our *ex vivo* and *in vivo* experiments revealed that *C. glabrata* isolates exhibiting a higher ECP level also had a higher burden and ECR rate and that the ECR rate was organ dependent. Notably, the ECR rate of intracellular *C. glabrata* cells using hMDM used in this study was significantly lower than that of THP1 in our previous study (18). Although *Mycobacterium tuberculosis-*infected THP1 and hMDMs exhibit similar effector functions, such as replication rate, phagocytosis, and cytokine production (33), our experience shows that, compared to hMDMs, *C. glabrata* cells have significantly higher intracellular replication and ECR rates inside THP1 macrophages when compared to hMDM. Understanding the basis of such differences deserves future studies. Furthermore, the media used in the present study were supplemented with L-glutamine, which has been shown to boost the effector function of hMDMs once encountered with *Aspergillus fumigatus* (34).

Given the importance of ECP level determination and the issues associated with the traditionally used CFU counting method, we were prompted to develop a rapid and precise method to identify ECP level in real time. We used a cell-impermeable dye that, in conjunction with flow cytometry, could distinguish the ECP levels. We hypothesized that the fraction of viable unstained cells should be proportional to the ECP levels determined by CFU counting. We initially employed a collection of clinical *C. glabrata* isolates displaying very similar MIC values, which belonged to various genotypes, and we simultaneously monitored the viable CFUs and viable unstained cells using counting and our methods, respectively. Although our assay identified multiple isolates displaying high ECP levels, CFU counting at 24 h revealed that some of those isolates exhibited unstable micafungin persistence, i.e., high survival at early hours but low survival at later hours, similar to the low ECP isolates. The same phenomenon was also identified for the clinical isolates of *C. glabrata* collected from Austria. We found that isolates displaying this unstable phenotype were *bona fide* high persisters and had a significantly higher ECR rate once challenged with micafungin in the context of *ex vivo* and *in vivo* models. This is consistent with our observations that CFU counting at 3 h, but not 24 h, could reliably predict the *in vivo* burden. Therefore, sole reliance on 24-h CFU counting could have resulted in misidentification of those isolates, and our SYTOX-based and 3-h CFU counting assays could better predict the *in vivo* trajectory of *C. glabrata* isolates exhibiting various ECP levels. Notably, we also found some degree of discrepancy between the two methods, and future *in vivo* studies are needed to determine which method could be used as the gold standard technique for the differentiation of HP and LP *C. glabrata* isolates.

Despite the importance of antifungal persistence level, traditional clinical practices only focus on internationally established antifungal susceptibility testing protocols, which are unable to determine the persistence level of fungal species once exposed to fungicidal drugs (14, 17, 18). Herein, we propose that echinocandin-susceptible *C. glabrata* isolates should be further stratified to potentially decrease the burden and minimize the emergence of antifungal resistance in infected patients. Notably, the high and low persistence threshold used in the present study are arbitrary (SYTOX^Neg^ threshold of approximately 28%), and the application of an extensive number of *C. glabrata* isolates is required to stratify the persistence level even further to low, intermediate, and high levels, similar to antifungal susceptibility patterns (susceptible, intermediate, and resistant). Accordingly, an extensive collection of isolates manifesting various ECP levels could be further subjected to *ex vivo* and *in vivo* models to better assess the implication of the ECP level difference with both burden and ECR rates. Second, the SYTOX^Neg^ threshold may vary depending on the flow cytometry device, i.e., model or manufacturer, and the user handling the samples, i.e., lab-lab variability, which necessitates conducting multicenter studies to reach a more conclusive threshold for proper defining HP and LP isolates. Third, the concentration of the drug used in this assay is a function of the MIC of the species of interest and, therefore, is subject to change and validation. For instance, *C. parapsilosis* has an intrinsically high MIC for echinocandins; therefore, the 1 µg/mL concentration proposed for *C. glabrata* in our study should be dramatically increased to higher concentrations, such as 16 µg/mL (35). Finally, the laboratory media used may also require further standardization if they are integrated and used in clinical practice. We noted that the addition of serum to YPD- and MOPS-treated RPMI allowed reliable differentiation of low and high persisters, which has also been suggested for inclusion in routine antifungal susceptibility testing to better predict *in vivo* outcomes (29, 30). Taken together, despite providing promising results, our assay requires standardization in the context of multicenter studies.

The next question to ask after considering the ECP level is how to devise an immediate therapeutic strategy to treat infections caused by high persisters using the currently available antifungal drugs. Given the favorable safety and the cidality profiles associated with echinocandins (36), one way to minimize the ECR emergence is to increase the concentrations of echinocandins to single or double high doses of 900 mg or even 1,400 mg (37). Some studies have shown that such high concentrations are tolerable and far from the maximum tolerable dosage once used in various patient populations (37). Nonetheless, various echinocandin concentrations to minimize the ECR rate still require *in vivo* mice models of GI tract colonization and systemic infection. Notably, concentrations within this range are inefficient in completely sterilizing the *C. glabrata* colonizing the GI tract. Second, using an *ex vivo* model, we previously showed that echinocandins are highly metabolically dependent, and therefore, alternating echinocandins with a non-metabolic-dependent antifungal, such as amphotericin B, resulted in a significant reduction in burden and ECR rate once tested in THP1-derived macrophage model (18). Nonetheless, the application of this alternating strategy still requires an *in vivo* model and real-life testing. Our group is actively studying this topic.

Overall, the tools presented in the present study show promise for high-throughput applications, such as anti-persister drugs and a library of mutant screening, which could ultimately not only aid in advancing our understanding of the biology of persisters but also assist in devising more efficacious anti-persister measures to curb the emergence of drug resistance in the clinic. Notably, some bacterial pathogens are extremely slow growing; therefore, it would be interesting to assess the applicability of our assay to infer the persistence level of clinical isolates as well as the isogenic library of mutants. Therefore, we envision the applicability of our assay beyond mycology.

## MATERIALS AND METHODS

### Strains and growth conditions

The six *C*. *glabrata* isolates used for our initial assessment of micafungin persistence and mouse infection model are listed in Table S1. The microbiological and clinical features of the Austrian clinical *C. glabrata* isolates used for confirmation are listed in Table S4. All *C. glabrata* isolates were grown on YPD agar plates (1% yeast extract, 2% dextrose, and 2% peptone) and incubated overnight at 37°C. The inoculums used for the experiments were grown in YPD broth and incubated overnight in a shaker incubator at 37°C. Our micafungin persistence measurements (*in vitro* and *ex vivo*) were carried out in cRPMI supplemented with RPMI, 10% heat-inactivated PBS, 1% L-glutamine, and 1% pen-strep.

Clinical *C. glabrata* isolates collected from Austria and Turkey were obtained from ongoing epidemiological studies and were reviewed by an ethical committee and assigned the following institutional review board codes: UN4926 and 20-2T/30, respectively.

### Antifungal susceptibility testing

Antifungal susceptibility testing was performed according to the CLSI M27 broth microdilution protocol (38) and included caspofungin, amphotericin B, fluconazole, micafungin, and anidulafungin (all from Sigma, St. Louis, MO, USA). Plates containing the cell suspension and drugs were incubated at 37°C for 24 h, after which the MIC values were determined visually. Fluconazole resistance was noted when the isolates had an MIC ≥64 µg/mL. Resistance to micafungin was defined when the MIC ≥0.25 µg/mL, whereas isolates with MIC ≥0.5 µg/mL were considered as caspofungin and anidulafungin resistant (39). The ECR genotype was determined using polymerase chain reaction amplification and Sanger sequencing of HS1 and HS2 of *FKS1* and *FKS2*, respectively, as described elsewhere (40).

### *In vitro* micafungin persistence measurement

Overnight-grown *C. glabrata* isolates in YPD broth (150 rpm and 37°C) were washed three times with PBS, and 10^7^ CFUs were transferred into cRPMI media and incubated for 2 h in a CO_2_ incubator without shaking. After 2 h, the *C. glabrata* cells were subjected to micafungin (0.125 µg or 1 µg/mL). The initial CFUs were determined before adding the drug. Plates containing drug-exposed *C. glabrata* isolates were incubated in a 37°C incubator for up to 72 h. The burden and killing dynamics were determined by normalizing the CFU at each timepoint against the CFU of the initial inoculum before drug exposure.

### Differentiation of primary human monocytes to macrophages

Primary human monocytes were isolated from leukopaks sourced from healthy donors attending the Massachusetts General Hospital following an established Institutional review board (IRB) protocol (2014P002377). Human leukocytes were collected from the leukopaks by overlaying on Ficoll (Thermo Fisher), followed by centrifugation, and red blood cells were lysed using RBC lysis buffer (Thermo Fisher). To isolate primary human monocytes, the collected leukocytes were subjected to EasySep Direct Human Monocyte Isolation Kit (STEMCELL Technologies). Monocyte viability and purity were assessed by flow cytometry using 7AAD and antibodies against CD14, CD16, and CD45. In all the cases, the viability and purity were >99% and ≥94% (double-positive CD45^+^-CD14^+^-CD16^−^), respectively. To differentiate monocytes into mature macrophages, monocytes were resuspended in cRPMI medium supplemented with 50 ng/mL human macrophage-colony stimulating factor (M-CSF) and seeded into 24-well plates (one million monocytes/well), after which the plates were incubated in a CO_2_ incubator at 37°C. After 4 days of incubation, the cRPMI medium was replaced with fresh cRPMI containing M-CSF (50 ng/mL), and plates were further incubated for another 3 days in a CO_2_ incubator at 37°C. On the day of infection (day 8), the cRPMI was replaced with fresh cRPMI without M-CSF, and fully mature primary human macrophages were infected with *C. glabrata* isolates.

### Infection of mature primary human macrophages with *C. glabrata*, *ex vivo* persistence, and ECR colony capture

On the day of infection, overnight-grown *C. glabrata* isolates were washed three times with PBS and counted, and 10^7^–10^5^ CFUs [Multiplicity of infection (MOI) of 10 and 0.1 yeast/1 macrophage) were added to micafungin-treated and untreated control wells, respectively. To eliminate the non-engulfed *C. glabrata* cells, all the wells were extensively washed with PBS at 2 h pi, fresh cRPMI with/without micafungin (0.125–1 µg/mL) was added, and plates were further incubated in a CO_2_ incubator at 37°C. At the designated timepoints, the macrophages were extensively washed and lysed by adding 1-mL cold water, and the lysates were subsequently transferred to YPD agar plates with or without micafungin (0.125 µg/mL) to capture ECR colonies and to assess burden, respectively. The intracellular burden was determined by normalizing the CFU at each timepoint against the CFUs of the pertinent untreated intracellular control 1 h pi. The ECR was determined by normalizing the number of ECR colonies against the burden of the pertinent treated samples. The *FKS* mutations were determined using the instructions provided elsewhere (40). Notably, micafungin-containing plates were incubated for up to 1 week and inspected daily to enumerate and collect the ECR colonies.

### Intracellular-induced persistence measurement

On the day of infection, overnight-grown *C. glabrata* isolates were washed three times with PBS and counted, after which 10^7^ CFUs (MOI of 10 yeast/1 macrophage) were added to each well. After 2 h of co-incubation, the macrophages were extensively washed with PBS and lysed by adding 1 mL of cold water. The precipitated *C. glabrata* cells were inoculated in cRPMI supplemented with micafungin (0.125 µg/ml), after which the cell suspensions were transferred to CO_2_ incubator at 37°C. The burden of the pertinent initial inoculum was determined before adding micafungin, and *C. glabrata* survival was determined by plating drug-exposed cells at designated timepoints. The dynamics of killing and burden were assessed by normalizing the CFU at each timepoint against the untreated control prior to adding the drug.

### Induction of systemic mouse infection and ECR colony capture

A systemic infection mouse model was carried out in Institutional Animal Care and Use Committee (IACUC)-accredited mouse facility at the Massachusetts General Hospital following an established, approved IACUC protocol (2017N000058). On the day of infection, the overnight-grown *C. glabrata* isolates were washed three times with PBS, and 6-week-old female C57BL/6 immunocompetent mice were infected with 200 µL of 10^7^ CFU of *C. glabrata* isolates via the tail vein route. Mice were categorized into multiple groups depending on the number of yeast isolates, and each major group had two more subgroups of treated and untreated. We included at least three mice in each subgroup (three treated and three untreated) for each timepoint, and in some cases, four mice were included in the treated subgroup. Micafungin was administered intraperitoneally (5 mg/kg), and treatment was initiated 1 day after infection and continued every other day throughout the experiment. Depending on the experiment, mice were dissected after 2, 4, and 6 doses of micafungin corresponding to days 5, 10, and 15 pi, respectively. Mice were euthanized prior to sacrifice, and both kidneys and the entire spleen and liver were collected, homogenized, and plated on YPD agar plates with/without 0.125 µg/mL of micafungin after extensive homogenization. The agar plates were incubated in a 37°C incubator for 72 h and up to a week. Burden was assessed by normalizing the CFU burden of treated mice at designated timepoints against the pertinent untreated control on day 5 pi. Burden results were presented as CFU per gram of each organ. The ECR was determined by normalizing the number of ECR colonies obtained from each organ at designated timepoints against the burden of the pertinent organ in a given mouse (the dilution factor was always considered for both burden and ECR rate determination). Notably, all YPD plates were treated with 0.5% pen-strep (Corning) to prevent potential bacterial contamination.

### Viability detection using flow cytometry

*C. glabrata* isolates exposed to micafungin were collected at designated timepoints and centrifuged (6,000 × *g*, 4 minutes, room temperature), the supernatant was decanted, and PBS containing SYTOX (Thermo Fisher, catalog no. S7020) was added to each sample and incubated at room temperature in the dark for 45 minutes, followed by centrifugation, decanting the supernatant (6,000 × *g*, 4 minutes, room temperature), adding 1-mL PBS, and subjecting the samples to flow cytometry (BD FACSCelesta). Notably, the SYTOX used in our experiment was in solution and was diluted in 1/2,000 PBS. We included untreated stained and unstained samples at all timepoints to precisely draw the gates. The gating strategy used is shown in Fig. 3C. The indirect viability, also called the persistence level, was calculated by normalizing the SYTOX^Neg^ fraction of treated samples against the untreated control, and the results are presented as percentages.

### Statistical analysis

The statistical analysis was carried out with SPSS software (v.24 for Windows; SPSS, Inc., Chicago, IL, USA) and *P*-values ≤0.05 were considered to indicate statistical significance. The Shapiro-Wilk test was used to determine the data distribution; therefore, we used the Mann-Whitney *U* test to determine the statistical significance of nonparametric data (such as intramacrophage data from Austrian *C. glabrata* isolates). Additionally, the nonparametric Kruskal-Wallis test was used to determine the average differences in the quantitative values among multiple independent groups (such as the liver burden of Austrian *C. glabrata* isolates on day 5). A parametric analysis of variance was used to determine the average differences in the quantitative values among multiple independent groups (such as the organ burden of Austrian *C. glabrata* isolates, except for the day 5 liver).

## SUPPLEMENTAL MATERIAL

Supplemental Figures mbio.00072-24-s0001.docx Figures S1 and S2.

Supplemental Tables mbio.00072-24-s0002.xlsx Tables S1-S4.

ASM does not own the copyrights to Supplemental Material that may be linked to, or accessed through, an article. The authors have granted ASM a non-exclusive, world-wide license to publish the Supplemental Material files. Please contact the corresponding author directly for reuse.
