# Supplementary material for: Echinocandin persistence directly impacts the evolution of resistance and survival of the pathogenic fungus Candida glabrata
Source: mBio. 2024 Mar 19;15(4):e00072-24. doi: 10.1128/mbio.00072-24 (PMC11005346; doi:10.1128/mbio.00072-24)
Supplement: Supplemental Figures — Figures S1 and S2. [file mbio.00072-24-s0001.docx]

Supplementary Figure 1. The individual killing curves of the echinocandin low persister isolates compared to those of the other clinical isolates. Two tailed *t*-tests were used for statistical analysis, and values ≤0.05 were considered to indicate statistical significance. *, **, and *** indicate P values ≤0.05, < 0.01, and < 0.001, respectively.


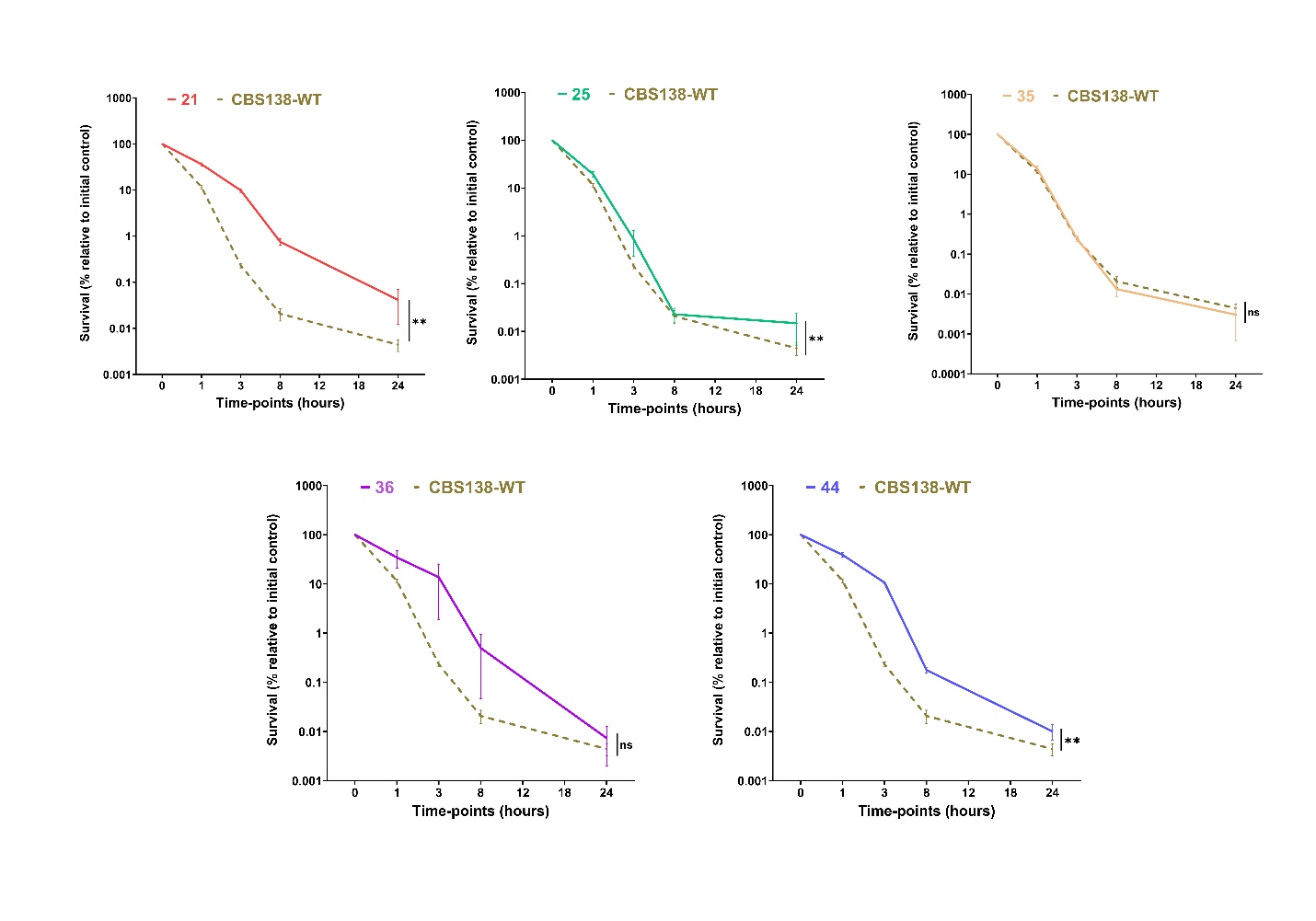


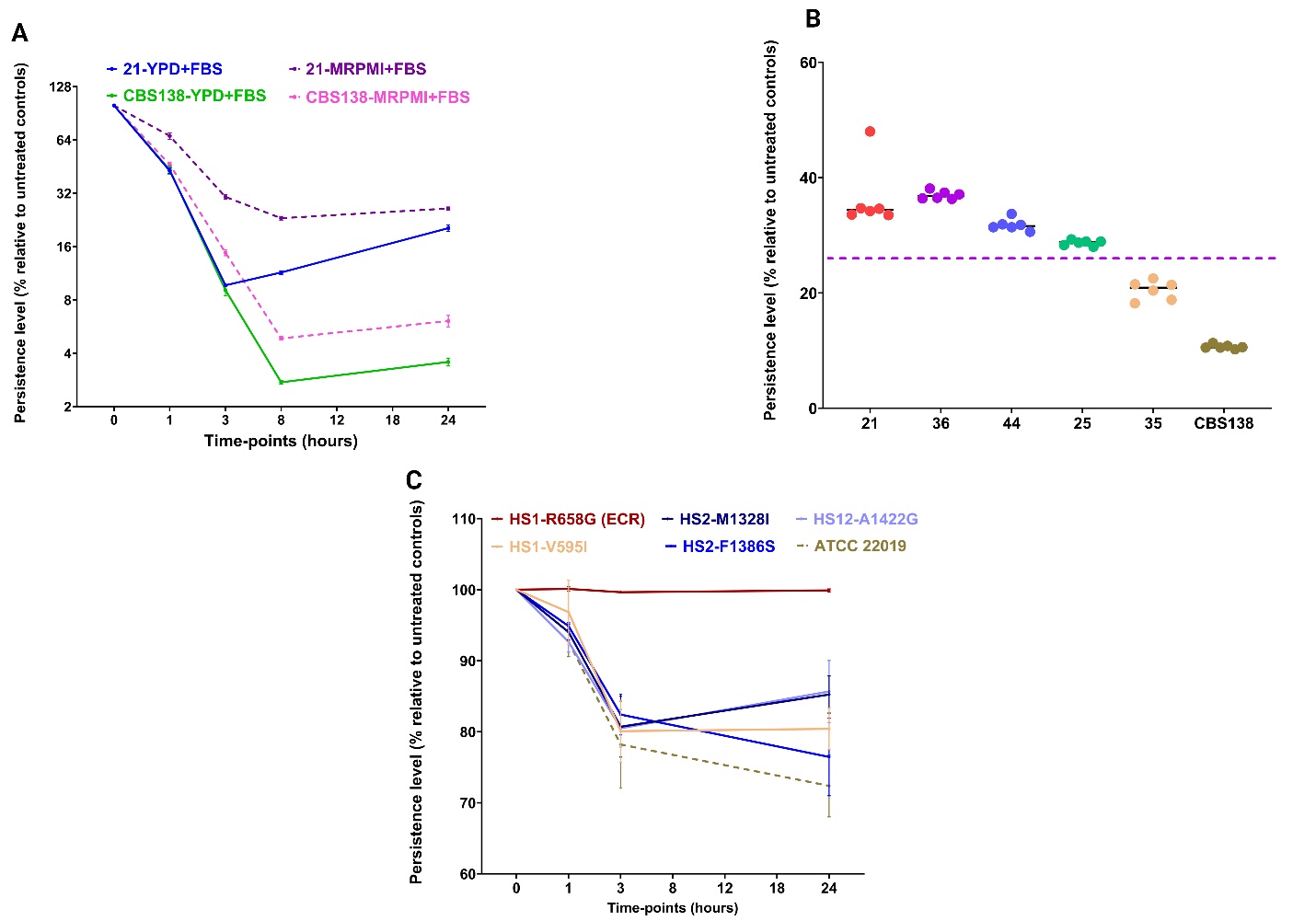


Supplementary Figure 2. 10% treated YPD and MOPS-treated RPMI cells reliably differentiated the low and high echinocandin-treated persister *C. glabrata* isolates (A). Using the threshold of 28% SYTOX^Neg^, our assay differentiated high- and lowpersisters (B). *C. glabrata* isolates were exposed to 1 µg/ml micafungin for 24 hrs, after which SYTOX staining and SYTOX^Neg^ fraction determination were performed via flow cytometry. Our SYTOX-based flow cytometry assay could distinguish the low and high echinocandin persister *C. parapsilosis* isolates (C).
